# Endoplasmic reticulum stress related genome-wide Mendelian randomization identifies therapeutic genes for ulcerative colitis and Crohn’s disease

**DOI:** 10.3389/fgene.2023.1270085

**Published:** 2023-10-04

**Authors:** Menglong Zou, Qiaoli Liang, Wei Zhang, Ying Zhu, Yin Xu

**Affiliations:** ^1^ The First Hospital of Hunan University of Chinese Medicine, Changsha, Hunan, China; ^2^ Zhuhai Second Hospital of Chinese Medicine, Zhuhai, Guangdong, China

**Keywords:** summary data-based Mendelian randomization, endoplasmic reticulum stress, ulcerative colitis, Crohn’s disease, integrative omics analysis

## Abstract

**Background:** Endoplasmic reticulum stress (ERS) is an important pathophysiological mechanism in ulcerative colitis (UC) and Crohn’s disease (CD). ERS-related genes may be influenced by genetic factors and intestinal inflammation. However, the role of ERS as a trigger or potential etiological factor for UC and CD is unclear, as the expression of ERS-related genes in UC and CD may be the cause or subsequent changes in intestinal inflammation. Here, we used a three-step summary data-based Mendelian randomization (SMR) approach integrating multi-omics data to identify putative causal effects of ERS-related genes in UC and CD.

**Methods:** Genome-wide association study (GWAS) summary data for UC (6,968 cases and 20,464 controls) and CD (5,956 cases and 14,927 controls) were extracted as outcome, and DNA methylation quantitative trait loci (mQTL, 1,980 participants) data and expression QTL data (eQTL, 31,684 participants) from the blood were obtained as exposure. The ERS-related genes were extracted from the GeneCards database, and then the GWAS summary data were integrated with the mQTL and eQTL data associated with ERS genes by SMR. Sensitivity analysis included two-sample MR analysis, power calculations, Bayesian co-localization analysis, and phenotype scanning were performed to evaluate the robustness of the results.

**Results:** A total of 1,193 ERS-related genes were obtained. The three-step SMR analysis showed that cg24011261 CpG site regulating *GPX1* expression was associated with a low risk of UC, whereas *GPX1* expression regulated by a combination of cg05055782, cg24011261, and cg05551922 CpG sites was associated with a low risk of CD. Sensitivity analysis further supports these findings.

**Conclusion:** This multi-omics integration study identifies a causal relationship between the role of ERS in UC and CD and suggests potential new therapeutic targets for clinical practice.

## 1 Introduction

Ulcerative colitis (UC) and Crohn’s disease (CD) are the two main subtypes of inflammatory bowel disease (IBD) (Hodson, 2016). The main differences between CD and UC are the varying areas of the digestive tract involved. CD is discontinuous and can involve any part of the digestive tract, but mainly the terminal ileum, whereas UC is continuous and involves only the large intestine and mainly the rectum (Abraham and Cho, 2009; Yamamoto and Ma, 2009; Khor et al., 2011). The main clinical symptoms of IBD are abdominal pain, diarrhea, hematochezia, and weight loss. IBD is becoming more prevalent worldwide, particularly in newly industrialized countries such as South Africa and America (Ng et al., 2017). It is worrying that the prevalence of IBD has exceeded 0.3% in many countries in Oceania, Europe and North America (Ng et al., 2017). Despite the growing number of treatment options, the treatment of IBD remains extremely challenging. Many factors are involved in the pathogenesis of IBD, but it is mainly attributed to immunological and genetic factors (Fugger et al., 2020). Unravelling the causality of these interactions may provide important insights into the pathogenesis of IBD and identify potential targets for treating the disease.

Endoplasmic reticulum (ER) is mainly responsible for facilitating protein folding and translocation to the appropriate destinations (Cubillos-Ruiz et al., 2017). The ER is the largest membrane network in the cell and its function is susceptible to extracellular stimuli and intracellular homeostasis (Cubillos-Ruiz et al., 2017). Various injuries, such as hypoxia and inflammation, disrupt ER function and lead to the accumulation of large amounts of unfolded and misfolded proteins, resulting in ER stress (ERS) (Cao, 2015). During this process, unfolded protein response (UPR) is triggered to attenuate ERS-induced cellular damage as well as to enhance cellular resistance to injury (Grootjans et al., 2016). However, UPR fails to compensate for cellular stress to restore ER homeostasis when ERS is persistent and severe, leading to activation of apoptotic signaling pathways and promoting apoptosis.

Disruption of the intestinal mucosal barrier function due to the interaction of multiple factors is a core event in the development and progression of IBD. Therefore, injury to intestinal epithelial cells (IECs), a primary component of the intestinal mucosal barrier, may play a critical role in this event. Research suggests that an imbalance of ERS in IECs promotes the progression of IBD (Grootjans et al., 2016). A study conducted by Xie et al. (2022) also observed a large amount of ERS in IECs of UC. A pilot proteomics study by Vieujean et al. found that ERS was associated with the progression of fibrous strictures in patients with CD (Vieujean et al., 2021). Although many studies have shown that the development of UC and CD is associated with ERS, no study has systematically and comprehensively investigated their potential causal relationship.

Mendelian randomization (MR) is an emerging method of epidemiological investigation that uses genetic variants as instrumental variables to assess whether there is a potential causal association between exposure and outcome (Burgess et al., 2019). In MR, random assignment of effect alleles largely avoids bias from unknown confounders such as lifestyle and environmental factors (Smith and Ebrahim, 2004). Genome-wide association studies (GWAS) investigate genetic associations between traits using single nucleotide variants (SNVs). Integrated multi-omics analysis is a novel approach in the post-GWAS era to investigate disease pathogenesis and identify key therapeutic targets (Do et al., 2017). Summary data-based MR (SMR) is an extension of the MR concept that integrates and analyses GWAS summary data with expression quantitative trait loci (eQTL) or DNA methylation QTL (mQTL), which were developed to prioritize causal variation mediated by gene expression or DNA methylation (DNAm) (Zhu et al., 2016; Wu et al., 2018). In this study, we used three-step SMR to assess the potential causal association between ERS-related genes and the two main subtypes of IBD. We then performed heterogeneity in the dependent instrument (HEIDI) test to investigate whether the causality was due to pleiotropy. Finally, multiple sensitivity analysis was performed to evaluate the robustness of the results.

## 2 Materials and methods

### 2.1 Study design

**FIGURE 1:**
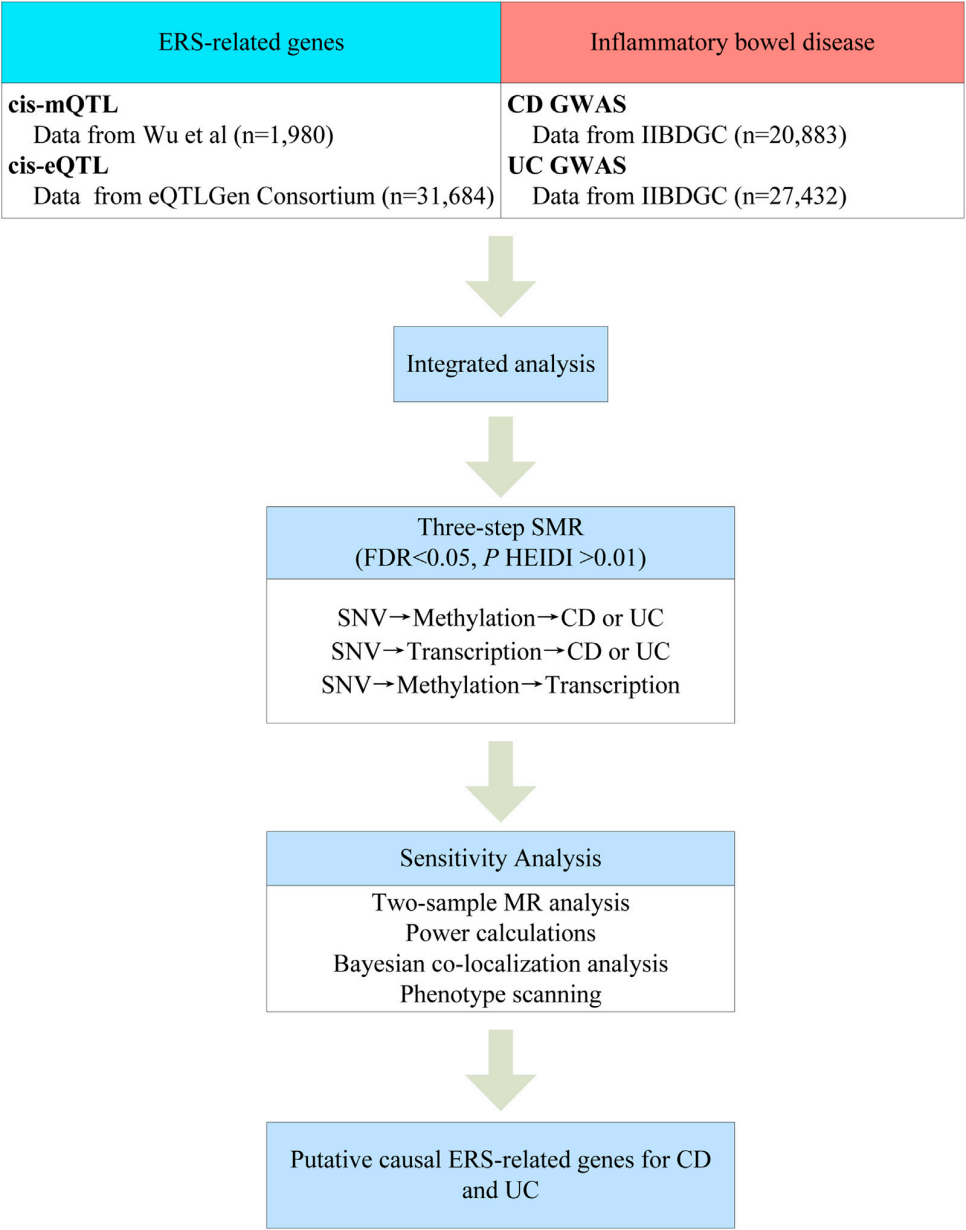
Workflow of the study.

Figure 1 summarizes the workflow of this study. First, we integrated GWAS summary data of the two major subtypes of IBD with the blood eQTL and mQTL summary data for analysis using the SMR method. Second, we identified potential causal ERS-related genes by three-step SMR. Finally, the robustness of the primary findings was further analyzed using two-sample MR analysis, power calculations, Bayesian colocalization analysis, and phenotype scanning.

### 2.2 Data source

The ERS-related genes were searched in the GeneCards database (https://www.genecards.org/) using the keyword “endoplasmic reticulum stress.” Only genes with relevance scores ≥7 were included in the analysis according to previous methods (Zhang et al., 2021; Guo and Liang, 2022; Xu et al., 2023). A total of 1,193 ERS-related genes were obtained (Supplementary Table S1). The blood cis-eQTL data and cis-mQTL data were obtained according to the methods previously reported in the literature (Jacobs et al., 2020; He et al., 2023). The cis-eQTL summary data for ERS-related genes were derived from the eQTLGen consortium, which collects genetic data on gene expression in 31,684 individuals from 37 datasets (Võsa et al., 2021). Every SNV-gene pair for which data was available in more than 1 cohort and with a SNV-gene distance of less than 1 MB was tested. Data were downloaded from the eQTLgen consortium website (https://www.eqtlgen.org/cis-eqtls.html). Notably, the downloaded data included non-significant SNVs. Therefore, a *P* < 5E-8 was set to extract SNVs of ERS-related genes from these data as instrumental variables. The cis-mQTL summary data were derived from a meta-analysis by Wu et al. (2018). Their study included two European cohorts: the Lothian Birth Cohorts (*n* = 1,366) and the Brisbane Systems Genetics Study (*n* = 614). These data are limited to DNAm probes with at least a cis-mQTL at *P* < 5E-8 and SNVs ≤2 MB from each DNAm probe. BESD format for these data was obtained from the SMR website (https://yanglab.westlake.edu.cn/software/smr/#mQTLsummarydata). The current study focused only on cis-mQTL summaries of ERS genes. GWAS summary data for UC were obtained from the international inflammatory bowel disease genetics consortium (IIBDGC), including 6,986 cases and 20,464 controls (Liu et al., 2015). Similarly, we also obtained CD GWAS summary data from the IIBDGC, which included 5,956 cases and 14,927 controls. The data used in this study were obtained from public databases that had already been ethically approved for the original study. Therefore, no additional ethical approval was required for this study.

### 2.3 Statistical analysis

The main analytical process of this study consisted of two stages: primary SMR analysis and sensitivity analysis. The SMR analysis provided the main results, while the sensitivity analysis tested the robustness of the results. We provide more detail on SMR analysis and sensitivity analysis in the Supplementary Methods.

Primary SMR analysis was conducted in three steps (1): SNVs as genetic instrumental variables, DNAm as exposure, and two major subtypes of IBD as outcome; (2) SNVs as genetic instrumental variables, ERS-related gene expression as exposure, and two major subtypes of IBD as outcome; (3) SNVs as genetic instrumental variables, DNAm as exposure, and ERS-related gene expression as outcome. Only the significance results from the first and second steps were included in the third step of the analysis. The identification of the final putative causal relationships was defined as (1) false discovery rate (FDR) < 0.05 in all three-step SMR; (2) *P* HEIDI >0.01 in all three-step SMR; and (3) the eQTL and mQTL should correspond to the same gene symbol. The results of SMR were estimated using odds ratios (OR), which was calculated as follows: OR = exp (β_SMR_), where exp represents the base of the natural logarithm.

Sensitivity analysis included two-sample MR analysis, power calculations, Bayesian co-localization analysis, phenotype scanning. Two-sample MR analysis was performed only on the causal results identified by SMR. We also performed power calculations using an online power calculator (https://sb452.shinyapps.io/power/) (Burgess, 2014). A power ≥80% was considered to have high statistical efficacy (Sanderson et al., 2022). Bayesian co-localization analysis was used to test whether GWAS summary data (including UC and CD) and QTL (including eQTL and mQTL) shared the same causal variable. The localization analysis was based on five hypotheses (H0, no association with GWAS or QTL within locus; H1, association with GWAS only; H2, association with QTL only; H3, association with GWAS and QTL but not co-localized; H4, co-localized GWAS and QTL) and the posterior probability of each hypothesis was assessed. Here, all SNVs within 100 kb upstream and downstream of the top SNV of the probe were extracted for co-localization analysis according to previous methods (Li et al., 2023). The posterior probability of H4 (PPH4) ≥ 0.5 was taken as evidence of co-localization of GWAS and QTL (Breen et al., 2019). Although PPH4 ≥ 0.8 is considered strong evidence for Bayesian co-localization, previous research has found that many loci with PPH4 ≥ 0.5 appear to be qualitatively consistent with the co-localization provided by PPH4 ≥ 0.8 (Dobbyn et al., 2018). We also performed phenotype scanning with PhenoScanner database (http://www.phenoscanner.medschl.cam.ac.uk/) to investigate the relationships of identified instrumental variants with other traits. The screening criteria for phenotype scanning were as follows: (1) the GWAS was derived from European ancestry; (2) the effect allele of the instrumental variable was consistent with our results; (3) the association of the instrumental variable with the trait met genome-wide significance (*P* < 5E-8); and (4) the absolute value of the effect size >0.01. In addition, we searched the GWASATLAS database (https://atlas.ctglab.nl/) for associations of putative causal ERS-related genes with traits.

The three-step SMR analysis and HEIDI test were performed using version 1.3.1 of the SMR software (https://yanglab.westlake.edu.cn/software/smr/#Download). Two-sample MR analysis were conducted using the “TwoSampleMR (version 0.5.6)” package of the R software (version 4.2.2). The co-localization analysis was performed using the “coloc (version 5.2.2)” R package.

For the three-step SMR analysis, the Benjamini–Hochberg method was used to adjust for false positives due to multiple testing. FDR <0.05 was defined as significant for SMR analysis. The two-sample MR analysis is a sensitivity analysis based on the SMR results. For two-sample MR analysis, *P* < 0.05 was defined as significant.

## 3 Results

### 3.1 SMR analysis for mQTL and GWAS data

The SMR test was used to extract SNVs and DNAm sites for 1,193 ERS-related genes from the blood mQTL data. A total of 4,100 CpG sites of ERS-related genes were obtained, which were associated with 1,321,603 SNVs. We then integrated mQTL for ERS-related genes with GWAS summary data for UC and CD, respectively. In concrete, the integration of mQTL with the UC GWAS summary data identified 34 DNAm probes corresponding to 17 ERS-related genes (SMR FDR <0.05 and *P* HEIDI >0.01). Meanwhile, integration of mQTL with CD GWAS summary data identified 75 DNAm probes corresponding to 37 ERS-related genes. Estimates of causality are expressed as β, and odds ratios of 1 standard deviation (SD) of ERS-associated gene expression level were obtained by calculating the expectation of β, and these results are presented in Supplementary Table S2.

Our results demonstrated that different instrumental variants regulating the same ERS-associated gene have different effects on DNAm levels. For example, one SD increase of *RNF186* methylation by rs3806308 was associated with 34.6% higher risk of UC (OR: 1.346, 95% CI: 1.240–1.461, SMR FDR = 3.97E-10), and conversely, one SD increase of *RNF186* methylation by rs12128452 was associated with 30.5% lower risk of UC (OR: 0.695, 95% CI: 0.626–0.772, SMR FDR = 2.51E-9). However, the effect of instrumental variants of *GPX1* on DNAm levels was synergistic. One SD increase of *GPX1* methylation by rs34293138 and rs111903592 was associated with 20.3% and 48.3% lower risk of UC, respectively. In summary, our analysis of the integration of UC GWAS and mQTL revealed the presence of 31 independent loci regulating methylation levels at 34 different CpG sites within 17 genes associated with ERS. Similarly, our examination of the CD GWAS summary data identified 63 independent loci that regulate methylation levels at 75 different CpG sites within 37 genes associated with ERS.

### 3.2 SMR analysis for eQTL and GWAS data

A total of 985 eQTL probes of ERS-related genes were obtained, which were associated with 498,776 SNVs. Integration of the eQTL with the UC GWAS and CD GWAS resulted in 11 and 16 ERS-related genes (Supplementary Table S3), respectively. In UC, it was found that two genes (*GPX1* and *CLN3*) exhibited a protective effect, while nine genes (*MAPKAPK2*, *IL24*, *BCL2L11*, *COL7A1*, *NFKB1*, *KDELR2*, *CTSB*, *JAK2*, and *STAT3*) were identified as risk factors. In CD, six genes (*SCARNA5*, *GPX1*, *ATF6B*, *BAD*, *RNFT1*, and *TMED1*) were associated with a low risk of disease and ten genes (*HSPA6*, *COL7A1*, *RFT1*, *ERAP2*, *JAK2*, *TMEM258*, *CCDC88B*, *TMED10*, *ATP2A1*, and *STAT3*) were associated with a high risk of disease.

### 3.3 SMR analysis for mQTL and eQTL data

It is well known that gene methylation affects gene expression. Therefore, we proceeded to explore the possible link between DNAm and gene expression by using DNAm as the exposure and transcripts as the outcome. After screening the results by SMR FDR <0.05 and *P* HEID >0.05, we obtained the regulatory relationships for the expression of five ERS-related genes regulated by nine DNA methylation CpG sites (Supplementary Table S4). For *GPX1*, there were four significantly associated methylation sites (cg07274523, cg05055782, cg24011261, and cg05551922), all of which were positively correlated with *GPX1* expression. For *OS9*, there were two significant methylation sites (cg18799399 and cg15848620) that exerted different regulatory effects on *OS9* expression. For *ATF6B*, *TMED10*, and *TMED1*, they are regulated by cg03317682, cg12149606, and cg01875838, respectively.

### 3.4 Multi-omics data integration

**FIGURE 2:**
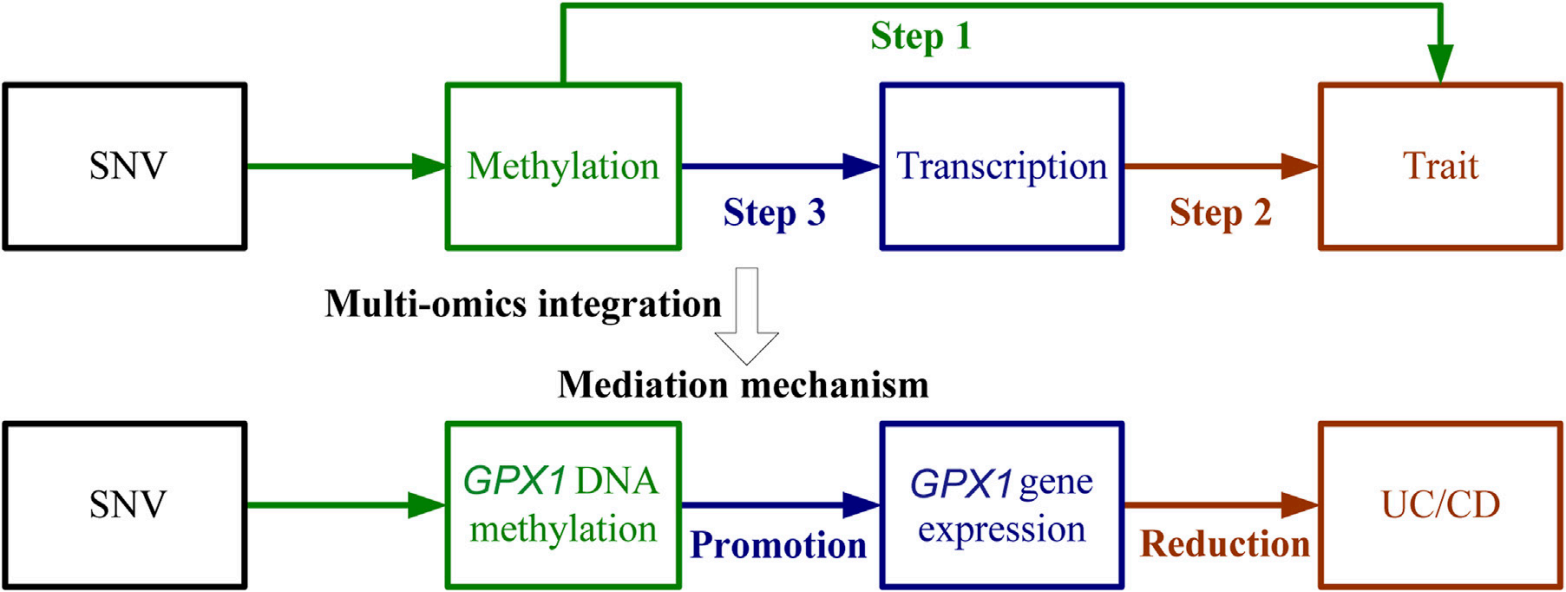
Schematic of integrative analysis of multi-omics data. The effects of DNAm on trait, DNAm on gene expression, and gene expression on trait are evaluated using the SMR and HEIDI method and integrated to identify potential mediation mechanisms in which a SNV exerts an effect on the trait by altering the DNAm level, which then regulates the expression levels of a functional gene. The detailed steps were (1) Use SMR and HEIDI to determine associations between DNAm and UC/CD; (2) Use SMR and HEIDI to determine associations between gene expression and UC/CD; (3) Use SMR and HEIDI to determine associations between DNAm and gene expression.

**FIGURE 3:**
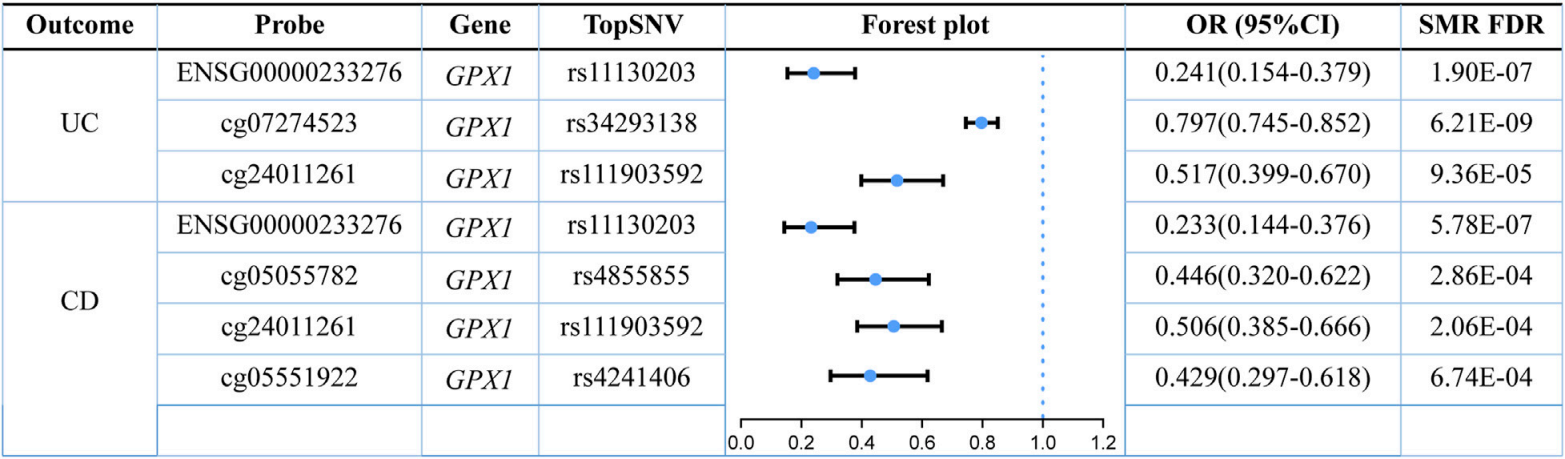
Forest plot of three-step SMR analysis results.

**FIGURE 4:**
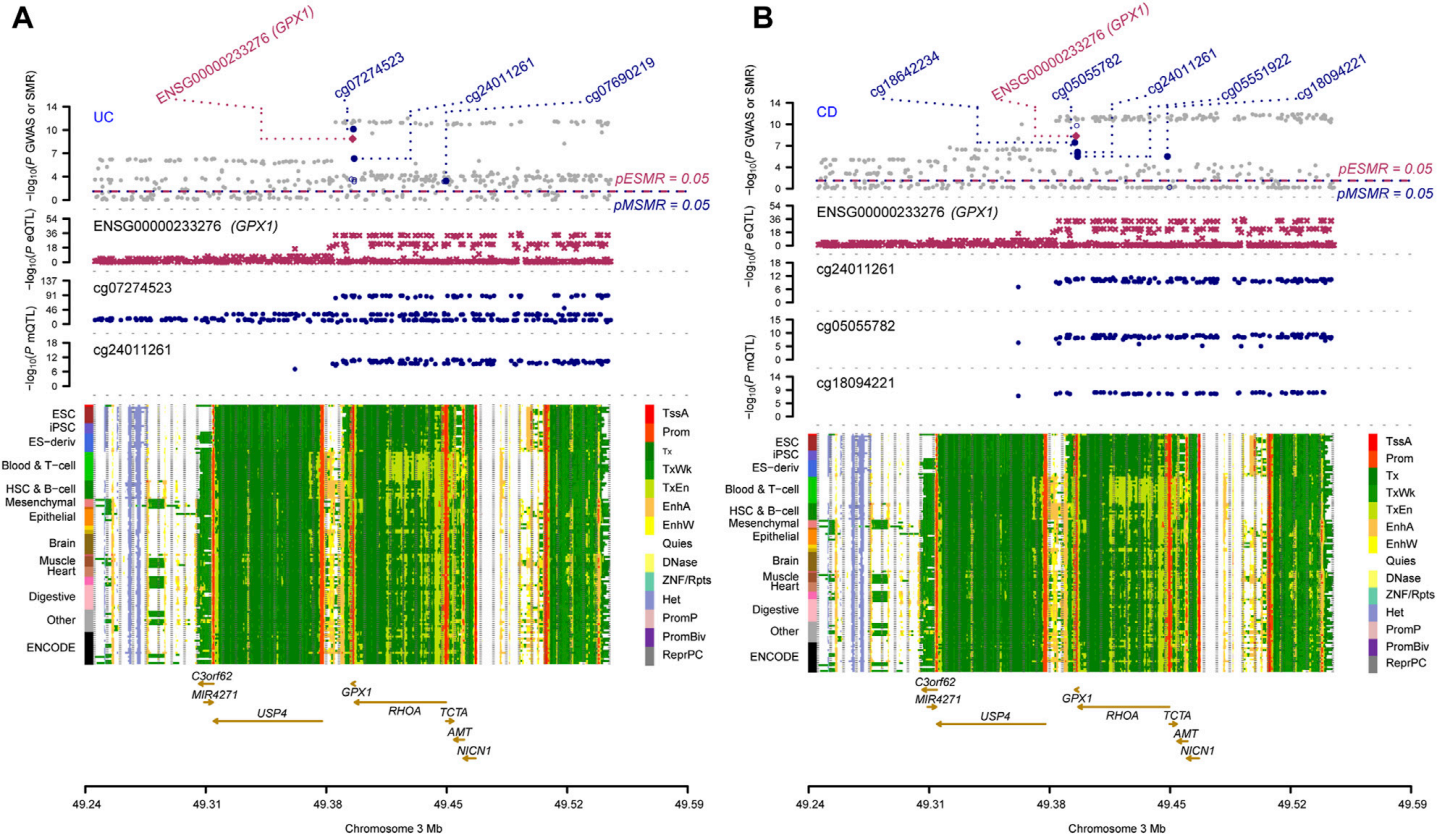
Results of SNV and SMR associations across mQTL, eQTL and GWAS at the *GPX1* locus. The top plot shows -log_10_ (*p*-values) of SNVs from GWAS. The red diamonds and blue circles represent -log_10_(*p*-values) from SMR tests for associations of gene expression and DNAm probes with trait, respectively. The solid diamonds and circles are the probes not rejected by the HEIDI test. The second plot shows -log_10_ (*p*-values) of the SNV associations for gene expression probe ENSG00000233276 (*GPX1*). The third plot shows -log_10_ (*p*-values) of the SNV associations for DNAm probes. The bottom plot shows 14 chromatin state annotations (indicated by colours) of 127 samples from the REMC for different primary cells and tissue types (rows). **(A)** multi-omics data integration for UC; **(B)** multi-omics data integration for CD.

Three omics results were integrated to identify causal effects of ERS-related genes in UC and CD. Based on the method for integrated analysis of multi-omics data proposed by Wu et al. (2018), we constructed a hypothetical model of the mediation mechanism: a SNV exerts an effect on the trait by altering the DNAm level, which regulates the expression levels of a functional gene (Figure 2). Four CpG sites, cg18799399, cg15848620, cg03317682, and cg12149606, were deleted because these sites did not show significance results in mQTL-GWAS SMR analysis. In addition, the cg01875838 site was discarded because *TMED1* did not show significant results in the eQTL-GWAS SMR analysis. In conclusion, the results of the integration analysis showed that *GPX1* methylation regulated by rs34293138 and rs111903592 influences *GPX1* expression, which is associated with low risk of UC, whereas *GPX1* methylation regulated by rs4855855, rs111903592, and rs4241406 influences *GPX1* expression, which is associated with low risk of CD (Figures 3, 4). For the cg07274523/cg24011261-*GPX1*-UC axis, β values for the causal association between cg07274523 (by the effect of the genetic variant rs34293138) to *GPX1* expression, cg24011261 (by the effect of the genetic variant rs111903592) to GPX1 expression, and *GPX1* expression to UC were 0.160, 0.450, and −1.422, respectively. For the cg05055782/cg24011261/cg05551922-*GPX1*-CD axis, β values for the causal association between cg05055782 (by the effect of the genetic variant rs4855855) to *GPX1* expression, cg24011261 (by the effect of the genetic variant rs111903592) to *GPX1* expression, cg05551922 (by the effect of the genetic variant rs4241406) to *GPX1* expression, and *GPX1* expression to CD were 0.407, 0.450, 0.428, and −1.458, respectively. As identified by the roadmap epigenomics mapping consortium (REMC), the four methylation probes (cg07274523, cg24011261, cg05055782, and cg05551922) that regulate GPX1 gene expression showed no significant differences in chromatin status across multiple tissues and cells (Figure 4), suggesting that there is no apparent tissue specificity to the SMR results. Furthermore, the top SNV in the mQTL-GWAS analysis of the cg07274523, cg05055782, cg24011261, and cg05551922 probes were 4, 15, 148, and 25 kbp distant from the top associated SNV in the eQTL-GWAS analysis of *GPX1*, respectively. Based on this evidence, we hypothesized that specific genetic variants lead to DNAm, which then upregulates *GPX1* expression. Theoretically, high expression of *GPX1* reduces the risk of UC and CD. Sensitivity analysis was performed using two-sample MR methods to verify the robustness of the SMR results. In cases where a single SNV was observed, the Wald ratio method was employed to assess causality. Conversely, when multiple SNVs were present, the inverse variance weighted method was utilized for the evaluation of causality. As expected, two-sample MR analysis supported our findings (Supplementary Table S5). The results of the power calculations also indicated that the data had a high statistical efficiency (Supplementary Table S6).

### 3.5 Bayesian co-localization analysis

**FIGURE 5:**
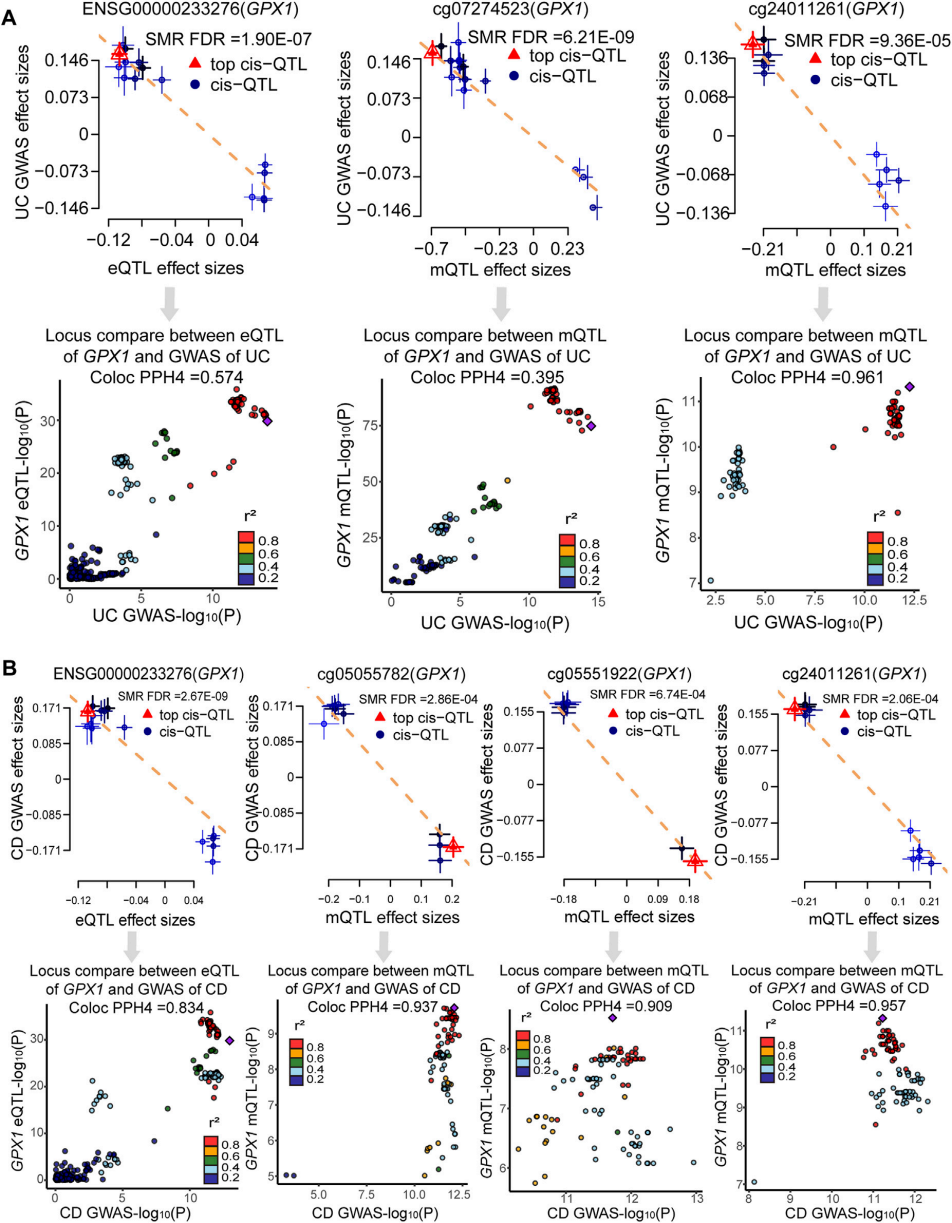
Result of SMR and co-localization analysis. **(A)** SMR and co-localization analysis for UC; **(B)** SMR and co-localization analysis for CD.

Bayesian co-localization analysis was used to rule out confusion due to linkage disequilibrium (LD). Bayesian co-localization results showed that *GPX1* expression (PPH4 = 0.574) and cg24011261 site (PPH4 = 0.961) shared the genetic variant with UC. However, our results did not indicate that cg07274523 site (PPH4 = 0.395) shared the same variant with UC (Figure 5A). Co-localization strongly suggests that *GPX1* expression (PPH4 = 0.834), cg05055782 (PPH4 = 0.937), cg05551922 (PPH4 = 0.909), and cg24011261 sites (PPH4 = 0.957) share genetic effects with CD (Figure 5B).

### 3.6 Phenotype scanning

To further validate the robustness of the results, we also searched for associations of the identified gene and genetic variants with other traits through the GWASATLAS and PhenoScanner databases, respectively. In the GWASATLAS database, we set *P* < 5E-8 to search for associations of *GPX1* with other traits (Supplementary Table S7). In addition to the study by Liu et al. (2015), the study by de Lange et al. (2017) and the study by Anderson et al. (2011) also showed that *GPX1* is associated with UC and CD, which is consistent with our findings. We searched the PhenoScanner database and found that rs11130203 and rs111903592 were associated with UC, whereas rs11130203, rs4855855, rs111903592, and rs4241406 were associated with CD (Supplementary Table S8). This also indicates the robustness of our findings.

## 4 Discussion

To our knowledge, this study is the first to identify putative causal ERS-related genes in UC and CD using a multi-omics integration approach and Bayesian co-localization. Integration of UC GWAS summary data and mQTL and eQTL for ERS-related genes prioritized one gene expression (*GPX1*) and two CpG sites (cg07274523 and cg24011261). However, the cg07274523 site did not pass co-localization analysis (PPH4 = 0.395), suggesting that the observed causality may be due to LD. Integration of CD GWAS and QTL data prioritized one gene expression (*GPX1*) and three CPG sites (cg05055782, cg24011261, and cg05551922). Our findings provide strong evidence for potential mechanisms linking the genetic locus, methylation and expression of *GPX1* to UC and CD.

**FIGURE 6:**
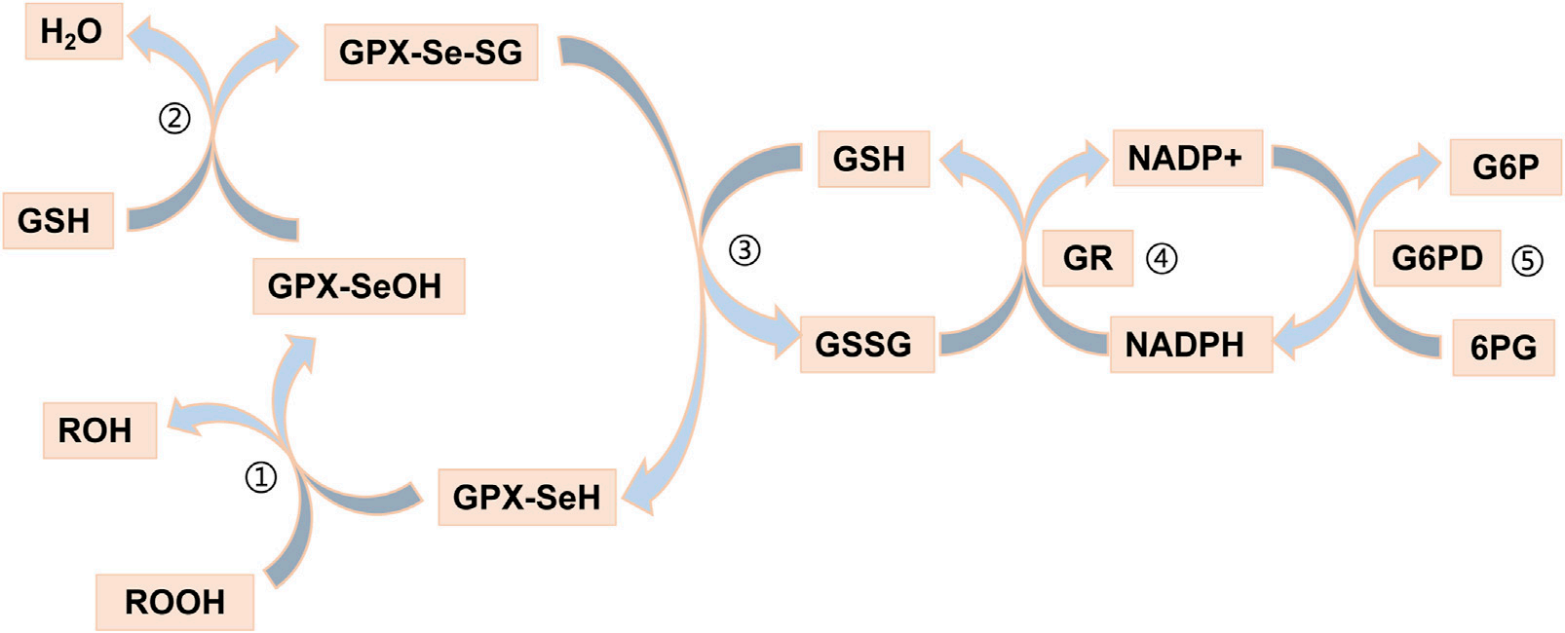
Reduction of hydrogen peroxide by *GPX1*. Step ①: The selenol of GPx-SeH (with -SeH representing the Sec active site) is oxidized to selenic acid (-Se-OH) by peroxide (ROOH); Step ②: The first GSH molecule reduces selenic acid (-Se-OH) to form glutathioneated selenol intermediate (-Se-SG) and releases a part of H_2_O; Step ③: The second GSH molecule continues to reduce the intermediate (Se-SG) to form oxidized glutathione (GSSG), while the activity of GPX neutrality returns to selenol (-Se-); Step ④: The GSSG is degraded to reduced glutathione (GSH) under the action of NADPH-dependent glutathione reductase (GR), while NADPH loses an electron to NAPD+. Step ⑤: Glucose 6 phosphate (G6P) reduces NAPD + to NADPH when circulating under the action of glucose 6 phosphate dehydrogenase (G6PD).

Recently, an increasing number of blood-based biomarkers have been used to predict the risk of developing UC and CD. We found a negative (protective) effect of *GPX1* expression with susceptibility to UC (OR_SMR_ = 0.241) and CD (OR_SMR_ = 0.233) by SMR analysis. Glutathione peroxidase 1 (*GPX1*) is a redox-active enzyme that mitigates cytotoxicity by catalyzing the reduction of organic hydroperoxides or H_2_O_2_ to the corresponding alcohols or water (Li et al., 2000). Selenocysteine (Sec) is an important component of the active site of *GPX1* (Flohe et al., 1973; Rotruck et al., 1973). The *GPX1*-catalysed process usually also requires the use of glutathione (GSH) as a reducing agent (Barbosa et al., 2017). Indeed, detoxification of peroxides by mammalian *GPX1* occurs via a bi-substrate ping-pong type enzymatic mechanism (Lubos et al., 2011). During peroxidase reduction, modification of the Sec active site is required to form a stable intermediate (Flohé et al., 1972; Flohe et al., 1973; Kraus et al., 1980). After reaction with peroxides, the active site of sec (-SeH) is oxidized to selenenic acid (Se-OH) (Kraus et al., 1980; Flohé et al., 2022). One of the GSH molecules then binds to selenenic acid and undergoes a reduction reaction to produce a glutathione selenol (Se-SG) intermediate (Kraus et al., 1980; Flohé et al., 2022). Another GSH molecule continues to bind to the intermediate and reduces it to oxidized glutathione (GSSG), a process that will restore the active site of sec (Kraus et al., 1980; Flohé et al., 2022). The GSSG is in turn degraded to GSH by NADPH-dependent glutathione reductase (GR) (Lubos et al., 2011). The process of reduction of hydrogen peroxide by *GPX1* is illustrated in Figure 6. *GPX1* is usually classified as an oxidative stress enzyme because of its ability to reduce hydroperoxides. Superficially, *GPX1* KO mice develop normally, but these mice are susceptible to injury due to increased oxidative stress and ERS when stimulated (Liu et al., 2021). Wild-type mice supplemented with selenium under the same conditions survived normally. A number of studies have been conducted to show that overexpression of reactive oxygen species (ROS) leads to inflammatory bowel disease due to damage to the intestinal mucosal barrier (Sun et al., 2023; Yao et al., 2023). The study by Huang et al. showed that scavenging ROS toxicity, improving cellular antioxidant capacity and alleviating cellular ERS by increasing *GPX1* expression could greatly rescue the cell death situation (Tao et al., 2023). The UPR signaling, which plays a major role in restoring ER homeostasis, is initiated by IRE1, PERK and ATF6 (Cantoni et al., 2022). The evidence suggested that PERK signaling and Nrf2 signaling were in crosstalk with each other (Harding et al., 2003). The activated PERK promotes Nrf2 phosphorylation and transcription (Cullinan et al., 2003). Activation of the Nrf2 pathway is known to help maintain the integrity of the intestinal epithelial barrier, involving mechanisms such as Zonula Occludens-1, claudin and MUC2 (Wen et al., 2019; Yuan et al., 2019; Wang and Wang, 2022). Interestingly, there seems to be a cycle, as activated Nrf2 increases *GPX1* and GSH expression to improve cellular stress resistance (Yin et al., 2015; Chen et al., 2019; Liang et al., 2021). In conclusion, *GPX1* has a protective effect against UC and CD, which could serve as a potential pharmacological target for the diseases.

This study has four strengths. Firstly, this study integrated multi-omics data to dissect GWAS signals and identify prioritization of gene expression and methylation. SMR was used as the primary analysis, and the results were integrated through a three-step approach, indicating strong evidence for their robustness. We conducted a comprehensive and systematic evaluation of the causal association between 1193 ERS-related genes and the two major subtypes of IBD. Secondly, summary data for UC and CD were extracted from the IIBDGC database, which defines them critically. Thus, the findings are free of bias due to the co-occurrence of the two subtypes. Thirdly, we performed sensitivity analysis using two-sample MR methods, Bayesian co-localization analysis and phenotype scanning, which further demonstrated the reliability of our results. Finally, the samples included only individuals of European ancestry, thereby reducing biases arising from diverse genetic backgrounds.

Our study also has limitations. Despite obtaining considerable GWAS summary data, there was a lack of protein quantitative trait loci data that were associated with ERS. Additionally, the available eQTL and mQTL datasets did not contain information on genetic variables present on the X and Y chromosomes. Secondly, our study exclusively focused on cis-eQTL and cis-mQTL data for ERS-related genes. However, whether trans-eQTL and trans-mQTL data might also influence the regulatory network to a large extent is unknown. Finally, it is still necessary to perform functional experiments to validate our findings. In addition, as ERS gene expression can be affected by a variety of factors, we believe that combining data from different molecular levels (e.g., metabolites and proteins) with GWAS data may lead to new discoveries in the future.

## 5 Conclusion

In conclusion, our three-step SMR analysis showed that cg24011261 CpG site regulating *GPX1* expression was associated with a low risk of UC, whereas *GPX1* expression regulated by a combination of cg05055782, cg24011261, and cg05551922 CpG sites was associated with a low risk of CD. This study identifies a causal relationship between the role of ERS in UC and CD and suggests potential new therapeutic targets for clinical practice.

## Data Availability

The original contributions presented in the study are included in the article/Supplementary Material, further inquiries can be directed to the corresponding authors.
