# Supplementary material for: Endoplasmic reticulum stress related genome-wide Mendelian randomization identifies therapeutic genes for ulcerative colitis and Crohn’s disease
Source: Front Genet. 2023 Oct 4;14:1270085. doi: 10.3389/fgene.2023.1270085 (PMC10583552; doi:10.3389/fgene.2023.1270085)
Supplement: Supplementary file 2 [file DataSheet1.docx]

**Supplementary Methods**

### Summary-data-based Mendelian randomization (SMR) analyses

SMR applied a two-step least-squares (2SLS) approach to estimate the effect size of an exposure on an outcome by using a genetic variant that is significantly associated with the exposure as an instrument (1). To better assess the causal effect of endoplasmic reticulum stress (ERS)-related genes on traits by SMR, SNVs with *P*_SNV-QTL_ < 5 × 10^−8^ were further extracted and included, and all SNVs with linkage disequilibrium (LD) r-squared > 0·90 or < 0·05 were excluded and the causal associations were calculated as:

β_SMR_ = β_SNV-GWAS_ / β_SNV-QTL_

β_SMR_ is calculated as the estimated effect size of ERS-related genes on UC or CD GWAS, where β_SNV-QTL_ is the estimated effect size of SNV on ERS-related genes (a genetic variant – exposure trait association) and β_SNV-GWAS_ is the estimated effect size of SNV on UC or CD (the same genetic variant – outcome trait association).

To control the rate of genome-wide type I error, we adjusted the SMR *P*-value with Benjamini-Hochberg method to account for multiple testing. A SMR FDR threshold of 0.05 was defined to assess the statistical significance of the association between ERS-related genes expression and DNA methylation and outcomes.

SMR also implemented the heterogeneity in dependent instruments (HEIDI) test to exploit if the observed association was due to vertical pleiotropy rather than the LD with the causal variant. The LD estimation was performed by using genomes of European ancestry obtained from the 1000 Genomes Project Consortium as reference (2). In addition, we checked the consistency of the alleles of each SNV between pairwise data sets (including the GWAS summary data, the mQTL summary data, the eQTL summary data, and the LD reference data). SPVs with allele frequency differences > 0.20 between any pair of the data sets are excluded from the SMR analysis. The results of the SMR are reliable if the excluded SNVs are less than 5% (1). In the three-step SMR of this study, less than 1% of SNVs were excluded at each step, suggesting that the results are informative. Association with the HEIDI test of *P* <0·01 was implied probably due to linkage rather than pleiotropy (the same variant controls both outcome and exposure independently) and should thus be discarded from the analysis (3).

### Sensitivity analyses

### Two-sample MR analyses

Sensitivity analyses were conducted after completing the primary SMR analyses with two-sample MR methods, including inverse variance weighting (IVW) and Wald ratio by using the “TwoSampleMR” R package. The Wald ratio estimate is the simplest way to obtain the causal estimate for a genetic variant which represented the exposure on the outcome when there was only a single genetic variant that was included in the analysis (4). The IVW was used when two or more genetic instruments were available.

### Co-localization Analysis

Co-localization is a method to assess the presence of a shared causal variant in the region for two traits. The basic hypothesis for colocalization in the same genomic location is:

H0: neither trait has a causal genetic variant

H1: only trait 1 has a causal genetic variant

H2: only trait 2 has a causal genetic variant

H3: both traits have a causal genetic variant, but not the same variant

H4: both traits share the same causal variant

HEIDI test is one of the co-localization methods that use external reference to estimate the LD. To refine the results, we performed another Bayesian test for co-localization of two traits using the coloc R package (<https://chr1swallace.github.io/coloc/>) to estimate the posterior probability of shared variants (5). For each leading SNV in the investigated GWAS database, all SNVs within 100 kb up and downstream of the leading SNVs were retrieved for co-localization analysis to analyze the posterior probability of H4 (PP.H4), with its default priors that the probability of a shared causal genetic variant for trait 1 (P1) and trait 2 (P2) is = 10^−4^ and the probability of a shared causal genetic variant across both traits is P12 = 5 × 10^−5^. Although the threshold for PP.H4 can be modified for multiple hypothesis testing, PP.H4 > 0·5 is the well-applied cut-off for the evidence of colocalization of the GWAS and QTL association.

### Phenotype scanning

We used the PheWAS dataset to examine the associations of putative causal ERS-related genes with traits in GWASATLAS (<https://atlas.ctglab.nl/PheWAS>). We also performed phenotype scanning with PhenoScanner database (http://www.phenoscanner.medschl.cam.ac.uk/) to investigate the relationships of identified instrumental variants with other traits. The screening criteria for phenotype scanning were as follows: (1) the GWAS was derived from European ancestry; (2) the effect allele of the instrumental variable was consistent with our results; (3) the association of the instrumental variable with the trait met genome-wide significance (*P* < 5×10^-8^); and (4) the absolute value of the effect size > 0.01.

**References**

1. Zhu Z, Zhang F, Hu H, Bakshi A, Robinson MR, Powell JE, et al. Integration of Summary Data from Gwas and Eqtl Studies Predicts Complex Trait Gene Targets. *Nature genetics* (2016) 48(5):481-7. Epub 2016/03/29. doi: 10.1038/ng.3538.

2. Genomes Project C, Auton A, Brooks LD, Durbin RM, Garrison EP, Kang HM, et al. A Global Reference for Human Genetic Variation. *Nature* (2015) 526(7571):68-74. Epub 2015/10/04. doi: 10.1038/nature15393.

3. Wu Y, Zeng J, Zhang F, Zhu Z, Qi T, Zheng Z, et al. Integrative Analysis of Omics Summary Data Reveals Putative Mechanisms Underlying Complex Traits. *Nat Commun* (2018) 9(1):918. Epub 2018/03/04. doi: 10.1038/s41467-018-03371-0.

4. Burgess S, Small DS, Thompson SG. A Review of Instrumental Variable Estimators for Mendelian Randomization. *Stat Methods Med Res* (2017) 26(5):2333-55. Epub 2015/08/19. doi: 10.1177/0962280215597579.

5. Giambartolomei C, Vukcevic D, Schadt EE, Franke L, Hingorani AD, Wallace C, et al. Bayesian Test for Colocalisation between Pairs of Genetic Association Studies Using Summary Statistics. *PLoS Genet* (2014) 10(5):e1004383. Epub 2014/05/17. doi: 10.1371/journal.pgen.1004383.
